# Emergence and Return Times in a Colonial, Cave‐Dwelling Bat: Age and Sex Differences Driven by Reproductive Cycle

**DOI:** 10.1002/ece3.71281

**Published:** 2025-05-04

**Authors:** Nicola J. Bail, Lindy F. Lumsden, Terry Reardon, Emmi van Harten, Paul Clissold, Thomas A. A. Prowse

**Affiliations:** ^1^ School of Biological Sciences The University of Adelaide Adelaide South Australia Australia; ^2^ Department of Energy, Environment and Climate Action Arthur Rylah Institute for Environmental Research Heidelberg Victoria Australia; ^3^ Cudlee Creek South Australia Australia; ^4^ Research Centre for Future Landscapes, Department of Environment and Genetics La Trobe University Bundoora Victoria Australia; ^5^ Humbug Scrub South Australia Australia

**Keywords:** cave‐dwelling bats, Chiroptera, emergence and return times, Miniopterus orianae bassanii, PIT‐tags, reproduction

## Abstract

The time that bats emerge and subsequently return from a colonial roost determines their maximum foraging period and influences their exposure to mortality risks. The order in which different age and sex cohorts emerge and return reflects variation in these cohorts' resource requirements. The critically endangered Southern Bent‐wing Bat (*Miniopterus orianae bassanii*) is an Australian insectivorous cave‐roosting colonial bat. Resource limitation is hypothesised to have contributed to its decline but may not affect all cohorts equally. We tagged and monitored 3462 wild Southern Bent‐wing Bats over 7 years with Passive Integrated Transponder technology. To infer resource requirements of different cohorts over the reproductive cycle, we estimated cohort‐specific peak emergence and return times and the frequency of nocturnal returns to the roost. The emergence and return behaviour varied with age, sex, and throughout the annual reproductive cycle. Although adult females and males behaved similarly during the non‐breeding period (winter), females emerged significantly (12–21 min) earlier and returned (27–62 min) later than males during pregnancy, lactation and weaning. Adult females were less likely than males to be detected overnight in the maternity roost while dependent young were present, suggesting that females prioritised maximising foraging over nocturnal nursing. When juveniles commenced flying, they delayed emergence until several hours after sunset (well after adults had departed the roost). During the 40‐day weaning period, they progressively emerged earlier, such that by the end of this period they emerged with the adults, then subsequently foraged for longer than adults over winter. Passive monitoring of emergence and return behaviour in colonial bats can provide valuable data to infer cohort‐specific resource requirements. Regular monitoring of a population's emergence and return times potentially allows for the early detection of changes in a resource requirements, and the use of PIT technology allows for the most vulnerable cohort(s) to be identified.

## Graphical abstract

We monitored 3462 wild Southern Bent‐wing Bats over 7 years with PIT‐tags and readers at key roosts to derive seasonal emergence and return times of age‐sex cohorts. Our findings indicate that cohorts whose resource requirements are exacerbated by stage of the reproductive cycle show significant changes in emergence and return times and may increase the risk these groups encounter.

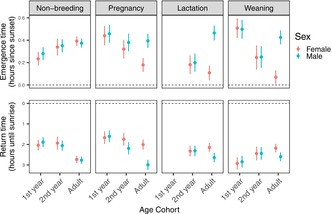

## 1 Introduction

For most bat species (Order Chiroptera), emergence from the roost signifies the onset of foraging activity. The timing of emergence and return determines a bat's available foraging period (Ramanantsalama and Goodman 2020). Many bat species form complex colonies comprised of different age and sex cohorts with variable energy, water and nutrient requirements (Carter and Wilkinson 2013; Kerth 2008). Within these colonies, the order in which cohorts emerge and return can reflect differences in their resource requirements (Arndt et al. 2018; Duvergé et al. 2000).

Emerging from roosts early and returning late can extend a bat's foraging period. Prey species of many insectivorous bats peak in abundance crepuscularly (Salvarina et al. 2018), which is thought to incentivise earlier emergence or later return for some insectivorous bat species (Jones and Rydell 1994). Colonial bats that emerge before the majority of their roost‐mates can also benefit from reduced intra‐specific competition for resources near the roost and at preferred foraging sites (Duvergé et al. 2000; Roeleke et al. 2018).

Colonial bats face proportionately higher risks during emergence from the roost and subsequent return than when roosting or foraging (Duvergé et al. 2000; Lima and O'Keefe 2013). Emergence in higher intensity light conditions facilitates predator attraction (Speakman, Lumsden et al. 1995), as bat colonies are often preyed on opportunistically during emergence (Feng et al. 2022; Lima and O'Keefe 2013). Some bat colonies delay emergence in response to predator cues (Thomas and Jacobs 2013; Welbergen 2006), but more often, emergence is delayed in response to high light intensity (Arndt et al. 2018; Speakman, Stone et al. 1995). Most bats behave in a manner consistent with equating activity in high light intensity with higher risk (Arndt et al. 2018; Rydell et al. 2021).

Resource limitation can modify the relationship between a species' usual emergence and return times and light intensity. For example, bats affected by White‐nose Syndrome emerge to forage diurnally (Bernard and McCracken 2017) as symptoms increase their resource requirements (Bernard et al. 2017; Hoyt et al. 2021). Reduced prey availability during drought also stimulates bats to emerge earlier and return later (Frick et al. 2012; Lee and McCracken 2001) and is implicated in reduced survival rates (van Harten et al. 2022a). The survival and foraging behaviour of individuals and cohorts with pre‐existing high resource requirements are generally the most affected by these resource‐restricting stressors (Jonasson and Willis 2011; Lee and McCracken 2001; van Harten et al. 2022a).

A bat's age, sex, reproductive status and developmental stage will affect its resource requirements and ensuing emergence behaviour. Generally, groups with high resource requirements emerge earliest and return latest, relative to roost‐mates. Lactating and post‐lactating females often emerge earlier, return later, and have lower survival rates than males and non‐breeding females (Lee and McCracken 2001; Petrželková and Zukal 2001; Reiter et al. 2008; van Harten et al. 2022a). This is likely due to the enormous resource investment required for lactation (Adams and Hayes 2008; Kurta et al. 1990; Kunz et al. 1995). As cyclical processes such as reproduction and development regularly alter the resource requirements of bat cohorts, it follows that foraging behaviour should differ between cohorts alongside these. Long‐term monitoring of bat colonies with Passive Integrated Transponders (PIT tags and associated readers) allows the typical order of emergence and return at a bat colony to be determined, and the behaviour of cohorts to be compared (Gillam et al. 2011; Jackson et al. 2022).

The critically endangered Southern Bent‐wing Bat (*Miniopterus orianae bassanii*) is an obligate cave‐roosting, aerial‐foraging subspecies restricted to south‐eastern Australia. Populations of these temperate bats congregate in large, mixed age‐sex maternity colonies over summer and occupy a number of associated non‐maternity roosts throughout the year (Dwyer and Hamilton‐Smith 1965; van Harten et al. 2022b). Closely related *Miniopterus* species reach reproductive maturity at 2 years of age (Dwyer 1966), and the available evidence for the Southern Bent‐wing Bat suggests a similar pattern. Southern Bent‐wing Bats have declined significantly across their range in recent decades, with the underlying causes unclear (Department of Environment, Land, Water and Planning 2020). Like many cave‐dwelling bats, Southern Bent‐wing Bats are threatened by roost degradation, disturbance, and the potential introduction of novel pathogens to their roosts (Department of Environment, Land, Water and Planning 2020; Tanalgo et al. 2022). Extensive wetland drainage and clearing of native vegetation have occurred within the Southern Bent‐wing Bat's foraging range since European colonisation, with intensive clearing and drainage in the study region occurring through to the 1990s (Department of Environment, Land, Water and Planning 2020; Department of Primary Industries and Regions South Australia 2023), which are likely to have contributed to the decline.

Energetic requirements are particularly high for the Southern Bent‐wing Bat, which regularly flies great distances to forage and move between roosts (van Harten et al. 2022b). Drought has been suggested to reduce survivorship of all cohorts of South Australian Southern Bent‐wing Bats, but had the greatest impact on lactating females, juveniles, and altricial pups (Bourne and Hamilton‐Smith 2007; van Harten et al. 2022a). Quantifying how resource requirements differ between Southern Bent‐wing Bat population cohorts could provide insights into the likely impact of threats on population structure and trends. If the extensive changes to native vegetation and wetlands in the subspecies' range are impacting this population, or cohorts within it, foraging patterns and associated emergence and return behaviour may reflect this.

We used data collected over a 7‐year period from a large sample (*n* = 3462) of free‐flying PIT‐tagged Southern Bent‐wing Bats in South Australia to investigate emergence and return behaviour across different cohorts of this population. We compared peak emergence and return times and nightly activity patterns between six age‐sex cohorts, and across key reproductive stages in the annual breeding cycle. Using these estimates as proxies for foraging time and resource requirements, we explore how resource needs differ between population demographics and reproductive stages at two Southern Bent‐wing Bat roost caves.

## 2 Materials and Methods

### 2.1 Study Sites

Southern Bent‐wing Bats were monitored at a maternity roost and an associated non‐maternity roost from 2016 to 2022. The maternity roost is located in Bat Cave, a multi‐chambered cave within the Naracoorte Caves National Park World Heritage Area in south‐east South Australia. Southern Bent‐wing Bats congregate here in the austral spring to form a maternity colony. Occupancy peaks in summer, when the site is occupied by approximately 28,000 adult bats, comprising both females and males of all ages (Hamilton‐Smith 1972). The cave entrance is a collapsed window measuring approximately 8 × 4 m, surrounded by vegetation of varying heights.

The non‐maternity roost is approximately 70 km from the maternity roost. Here the cave entrance is in a large doline (a funnel like depression in the ground), and measures approximately 6 × 2 m. The cave is situated on a dairy farm and is surrounded by cleared pastures. Southern Bent‐wing Bats are known to move regularly between this site and the maternity roost (van Harten et al. 2022b).

### 2.2 Automated Fly‐Out and Fly‐In Counts at the Maternity Roost

A monitoring program was established at the maternity roost in late 2021 to estimate Southern Bent‐wing Bat population size each night. An infra‐red camera and illuminators are installed outside the cave, which film emergence and return from dusk until dawn. The Southern Bent‐wing Bat is the only bat that roosts in this cave. Population estimates are derived using the bespoke *N2* software package specifically created to tally emerging and returning bats within each minute of every sampled night at this site. This system provides information about the emergence and return times of the whole aggregation, complementing PIT‐tag data that provides additional information on the behaviour of age and sex cohorts. We primarily used these data to evaluate the accuracy of emergence and return estimates from the PIT antenna positioned inside the cave (see below).

### 2.3 PIT‐Tagging and Detection

A total of 2966 Southern Bent‐wing Bats were trapped and PIT tagged at the maternity cave from 2016 to 2018, as described in van Harten et al. (2019, 2020). We PIT tagged an additional 496 bats in January and February 2022, following the same method. Bats were trapped throughout the night, from dusk until just before dawn, with two‐bank Austbat harp traps (Faunatech/Austbat, Mount Taylor). Bats were sexed based on external genitalia and aged, with juveniles (< 3 months old) classified based on the shape of metacarpal phalangeal joints and presence of cartilage in these joints (Brunet‐Rossinni and Wilkinson 2009). Prior to tagging, bats were scanned for existing PIT tags, then the forearm length and mass of each bat were recorded. Sample sizes were similar for males and females, with marginally more juveniles than adults tagged in later years (795 AF, 671 AM, 969 JF, 1027 JM; Table S1).

In 2022, the PIT tags used were Biomark APT12, with Biomark HPT12 PIT tags used from 2016 to 2018 (van Harten et al. 2019). Both tags are 12 mm glass encapsulated microchips with a high read range on stationary PIT readers (Karltek 2021 pers. comm.). Tags were inserted subcutaneously to rest between the scapulae, with the insertion site sealed with tissue glue to minimize tag loss (van Harten et al. 2020, 2021). PIT‐tag placement was checked before releasing the bats. This technique is associated with high tag retention, with PIT‐tag loss of 2.7% in a similar‐sized bat, *Chalinolobus gouldii*, all recorded within 2 months of tagging (van Harten et al. 2021).

A Biomark IS1001 PIT reader system was installed in the maternity cave in January 2016. This system consists of a tag reader, data logger, and a 15 m flexible cord antenna, as described in van Harten et al. (2019). The system was installed in the closest suitably sized cave passage, which is *c*. 100 m from the entrance (van Harten et al. 2019) and *c*. 30 m from the maternity chamber (Dwyer and Hamilton‐Smith 1965). An identical system was installed at the entrance of the non‐maternity roost in April 2017 (van Harten et al. 2019). PIT‐tag detections are recorded in real time by these readers, although power outages and high electromagnetic interference have caused temporary loss of data for short periods (van Harten et al. 2019).

All data processing and statistical analyses were performed in *R* (v. 4.2.2) software for statistical and graphic computing (R Core Team 2022).

### 2.4 Classification of Age‐Sex Cohorts and Reproductive Periods

PIT‐tagged individuals (detected from 2016 to 2022) were classified into cohorts based on their sex and age at capture. The assumed birth date of first‐year individuals was November 24th, the mean date of mass birthing at the maternity roost during the study period (T. Shortt 2023, pers. comm; van Harten et al. 2022b). Juveniles were classified as first‐year males or females from their initial capture date until 24/11/[capture year], then reclassified as second‐year males or females for the next 12 months. Second‐year male and female cohorts were separated from the adult cohorts to examine the effects of age and reproductive maturity separately. After the second year of life, bats were then reclassified as adult males or females on 24/11/[capture year + 1]. Adults and second‐year individuals without tags could not be distinguished on capture, so all non‐juveniles were excluded from analysis in their capture year and classified as adults on November 24th of the following year.

Based on current and past observations of birthing behaviour (Crichton et al. 1989; van Harten et al. 2022b), we defined four key stages of the annual reproductive cycle of Southern Bent‐wing Bats: the non‐breeding, pregnancy, lactation and weaning periods (Table 1).

**TABLE 1:** Key reproductive periods defined in this study for the Naracoorte Caves population of Southern Bent‐wing Bats, and their durations and justifications for defined dates. First‐year individuals are referred to as pups before becoming volant, and as juveniles when volant but still distinguishable from adults.

Allocated period	Duration	Justification
Non‐breeding Mating and then retarded foetal development; first‐years weaned	19th February–12th September	Mating and fertilisation occur in late autumn to early winter (Crichton et al. 1989), with blastocyst implantation and subsequent foetal development delayed. Embryogenesis has resumed by 12th September (Crichton et al. 1989, Bail pers. obs.)
Pregnancy Pregnancy developing normally, females not lactating	13th September–24th November	Embryogenesis continues. Mean date of mass birthing from 2016 to 2022 was November 24th (van Harten et al. 2019, pers. obs.)
Lactation Females have given birth and pups are fully dependent on milk	25th November–9th January	Females have given birth, pups are not yet volant or foraging outside the roost (van Harten et al. 2022a, 2022b)
Weaning First‐years volant and weaning off milk	10th January–18th February	First‐years are volant inside, and starting to emerge from, the cave in early January, progressively increasing foraging behaviour and are weaned and independent by 18th February (van Harten et al. 2022a, 2022b)

### 2.5 Emergence and Return Times

We derived sunrise and sunset times for each monitoring night at each roost using the bioRad package (Dokter et al. 2019). Daytime observations between 1 h after sunrise and 1 h before sunset were excluded from analysis, as bats detected in this period were presumed to be moving within the cave.

The emergence and return time of each cohort was estimated separately for each night of PIT‐tag monitoring (*n* = 1807 nights at maternity roost, 1256 at non‐maternity roost). We defined each cohort's emergence (and return) time as the peak detection period of individuals in a cohort before midnight, in units of decimal hours since sunset, and return as decimal hours until sunrise.

To calculate emergence and return times, we tallied the number of unique tagged individuals from each cohort that were detected within each 5‐min interval over the course of each night. To smooth out short‐term fluctuations, we then used a moving‐average smoother to calculate the mean detection count over every three consecutive (5‐min) intervals. The 5‐min interval corresponding to the maximum moving‐average value was deemed the emergence (peak detections before midnight) or return (peak detections after midnight) time for that cohort on that night.

We analysed emergence and return times at each roost separately, with generalised additive models (GAMs) using the package ‘mgcv’ (Wood 2011). To compare differences in emergence and return times between cohorts and reproductive periods, we included these fixed factors and their interaction as explanatory variables (Table 2). We also accounted for changes in these times due to the annual progression with a cyclic penalised cubic regression spline on the day of the year and included a random effect of year to consider unexplained variation between years. To account for improved accuracy of nightly peak emergence and return times when more bats were detected, we weighted the GAM model likelihoods for each estimate by the associated moving‐average peak count of bats detected (as derived above). Tukey's tests were used to compare the estimated mean emergence and return times in each period between cohorts at each roost, and between periods for each cohort.

**TABLE 2:** Details of the GAM model fit for different response variables for emergence patterns of southern bent‐wing bats. The structural goodness‐of‐fit of models for the maternity roost (MR) and non‐maternity roost (NMR) was assessed with pseudo‐*R* ^2^ values and the percentage of deviance explained. Spline functions for a continuous explanatory *variable* are denoted as *s* (*variable*). Period represents reproductive period, and cohort represents age‐sex cohort.

Cohorts	Response variable	Explanatory variables	Family	*R* ^2^		Deviance explained	
				MR	NMR	MR	NMR
All	Emergence time (Hours since sunset)	(1|Year) + Period + Cohort + Period:Cohort + *s* (day of year)	Gaussian	0.345	0.240	34.8%	25.0%
	Return time (Hours to sunrise)	(1|Year) + Period + Cohort + Period:Cohort + *s* (day of year)	Gaussian	0.332	0.496	34.5%	51.6%
All	Probability of detection	(1|Year) + Period + Cohort + Period:Cohort + *s* (time of night)	Binomial	0.382	0.192	44.0%	29.8%

To explore changes in first‐year bats' emergence and return times throughout the weaning period, relative to other cohorts, emergence and return times were also separately estimated for all first‐year bats (regardless of sex) and older cohorts throughout the 40‐day weaning period at the maternity roost. The emergence and return times of first‐year bats and all other cohorts over this period were analyzed with separate GAM models that included day of the weaning period and a random effect of year (Table S2). These models were only fitted to data from the maternity roost, as all first‐year bats are at this location during the weaning period.

Emergence times are measured in minutes since sunset; return times are measured in minutes until sunrise.

### 2.6 Population‐Level Emergence and Return Times From the PIT and N2 Datasets

Peak emergence and return times were also estimated for all tagged bats, regardless of cohort, to estimate the colony‐level emergence and return times at the maternity roost on a sample of nights when PIT monitoring and *N2* monitoring were both being conducted (199 nights compared for emergence times, 147 nights compared for return times). Peak emergence and return times were then derived from the *N2* monitoring data based on detections of all exiting and entering, using the method detailed above. We used paired *t*‐tests to compare in‐cave (PIT monitoring) and outside‐cave (*N2* monitoring) estimates for emergence and return time over this period, and within key reproductive periods (Table 1).

### 2.7 Overnight Activity Patterns

To investigate how often bats return to their roosts throughout the night in each reproductive period, we modelled the probability of recording PIT‐tagged individuals from each cohort over the course of each night, for each reproductive period (Table 1). First, we calculated the total number of bats recorded on the reader from each cohort on each night (i.e., from an hour before sunset until an hour after sunrise). Second, we tallied the number of bats of each cohort recorded in each 5‐min interval over the course of each night. Using the number of individuals of a cohort recorded within each 5‐min interval relative to the number of bats from that cohort recorded that night, we modelled the probability of a bat being detected within each interval over the course of the night using a binomial generalised additive model (GAM) and package mgcv (Wood 2011). Cohort and reproductive period were included as explanatory fixed factors, along with a random effect of year. To investigate changes in nightly activity patterns between groups over the course of a night, we also included time since sunset as an explanatory variable within this model, using a thin‐plate regression spline fitted within each cohort and period combination (Table 2).

## 3 Results

### 3.1 Comparison of N2 and PIT Emergence/Return Estimates at the Maternity Roost

**FIGURE 1:**
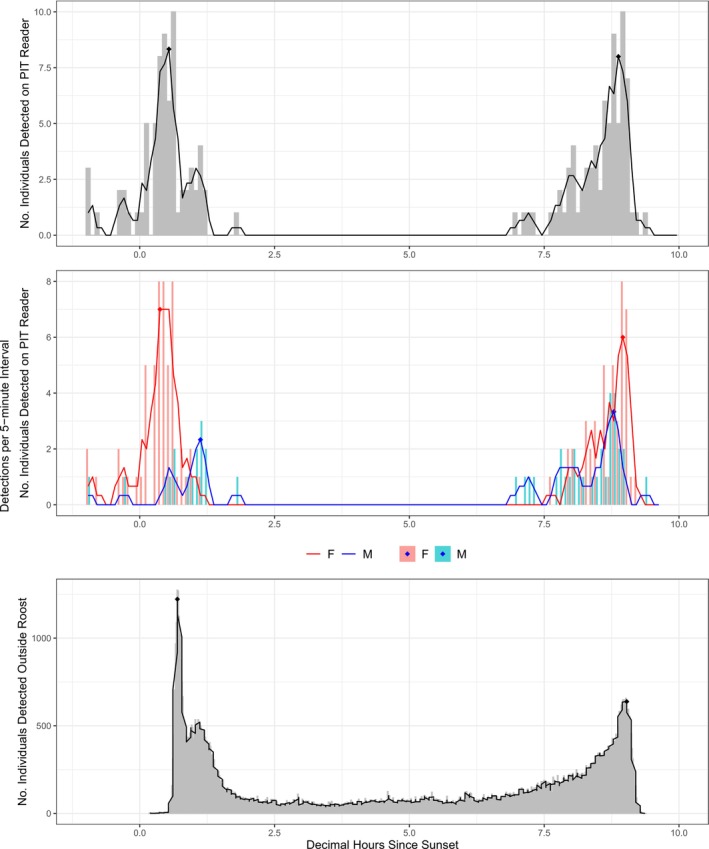
An example of emergence and return patterns on January 14, 2022 at the maternity roost, estimated with two methods. Estimates of peak emergence and return times (points) have been derived from the in‐cave PIT‐tag reader for all present bats (top panel), male and female bats (middle panel) and from video footage of emergence and return using the *N2* software at the entrance of the cave (bottom panel). Shaded bars signify the raw number of detections in each 5‐min interval, lines represent the moving average of these values (over 3 intervals). In the middle panel, female detections are presented in red and male detections in blue.

Emergence times estimated from PIT‐tag data (i.e., from detections by the in‐cave antenna) preceded emergence times derived from *N2* data (i.e., from monitoring outside the cave). Across the 2021–2022 time period, emergence times derived outside the cave occurred, on average, 16.9 min(m) (95% CI = 9.8 m, 23.8 m) later than emergence times derived from the in‐cave reader. Return time estimates were more similar between methods. Although absolute emergence time estimates differed between the two monitoring systems, activity patterns were largely consistent between them. For example, bimodal emergence peaks observed outside the cave with the *N2* system were also observed on the PIT‐tag reader, with the latter system also revealing differences between cohorts (Figure 1).

### 3.2 Cohort‐Specific Emergence and Return Times From PIT Monitoring

#### 3.2.1 Maternity Roost

**FIGURE 2:**
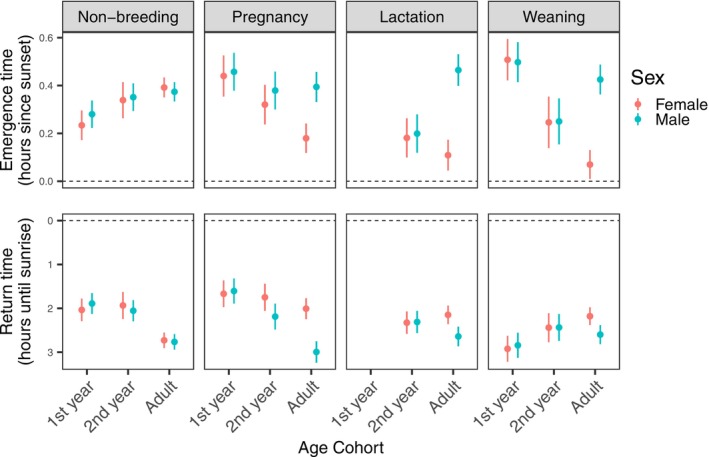
Estimated mean emergence and return times of southern bent‐wing bat cohorts in each reproductive period at the maternity roost, predicted by peak emergence and return time models. Line length indicates 95% confidence intervals for each estimate. First‐years are reclassified as second‐years at the beginning of the lactation period, so there are no volant first‐year cohorts during this period.

During the non‐breeding period, the emergence times of adult females and males (denoted below as AF and AM, respectively) did not differ significantly (estimate [95% confidence interval] = AF: 55.3 min since sunset(m), [49.8 m, 62.3 m]; AM: 54.1 m, [47.3 m, 66.1 m]), nor did their return times (AF: 214.2 min until sunrise(m), [184.8 m, 243.6 m]; AM: 215.4 m, [187.2 m, 244.2 m]). However, both adult cohorts emerged later than first‐year cohorts and returned earlier than all younger cohorts during this period (Figure 2 and Tables S3, S4).

Relative to all other reproductive periods, adult females at the maternity roost emerged later (Tukey's tests, all *p* < 0.001) and returned earlier (all *p* < 0.002) during the non‐breeding period (Figure 2). Adult females emerged before adult males and returned after them in all three periods involved in reproduction and rearing pups (i.e., during pregnancy, lactation and weaning). This difference was most marked during the lactation and weaning periods, where adult females emerged 21 min 54 s and 21 min 29 s hours earlier than adult males, respectively (Figure 2 and Table S3). Furthermore, during the weaning period, adult females emerged earlier (34.7 m [26.9 m, 42.7 m] after sunset) than all other cohorts and returned later (181.8 m [149.4 m, 214.2 m] before sunrise) than all but second‐year cohorts. Although adult males' emergence and return time estimates were similar between all periods in the study, they emerged later and returned earlier than all cohorts during the lactation period (Tables S3, S4) and returned earlier in the pregnancy period (Figure 2).

Second‐year females and males (denoted below as 2YF and 2YM, respectively) initially (over summer) had comparable emergence and return time estimates; both emerged earlier (2YF 11.3 m earlier, 2YM 10.5 m earlier) and returned later (2YF 25.4 m later, 2YM 26.3 m later) than adult males during the lactation period, and both emerged after adult females (2YF 10.4 m later, 2YM 14.6 m later) and before first‐year cohorts (Table S3) in the weaning period (Figure 2). By the pregnancy period, the emergence and return times of second‐year cohorts began to resemble those of adult cohorts of the same sex. Second‐year females and adult females' emergence times did not significantly differ in this period, although adult females emerged significantly earlier than all other cohorts (all *p* < 0.001). Second‐year females also emerged 8 m 38 s before adult males in this period (*p* < 0.001), whilst second‐year and adult males' emergence times did not differ. Second‐year males returned before first‐year cohorts in this period, as did adult males, but second‐year females and adult females did not.

At the maternity roost, first‐year females and males (denoted as 1YF and 1YM, respectively) did not differ from each other in estimated emergence or return time. During the non‐breeding period, first‐year females and males emerged earliest and returned latest (1YF: 169.2 m, [137.4 m, 201 m]; 1YM: 159.6 m, [128.4 m, 190.8 m]) (Figure 2). Then in the pregnancy period, first‐year females and all male cohorts emerged after adult females (all *p* < 0.001), and both first‐year cohorts returned after adult males (1YF 81.4 m later, 1YM 62.2 m later) and second‐year males (1YF 34 m later, 1YM 34.8 m later).

**FIGURE 3:**
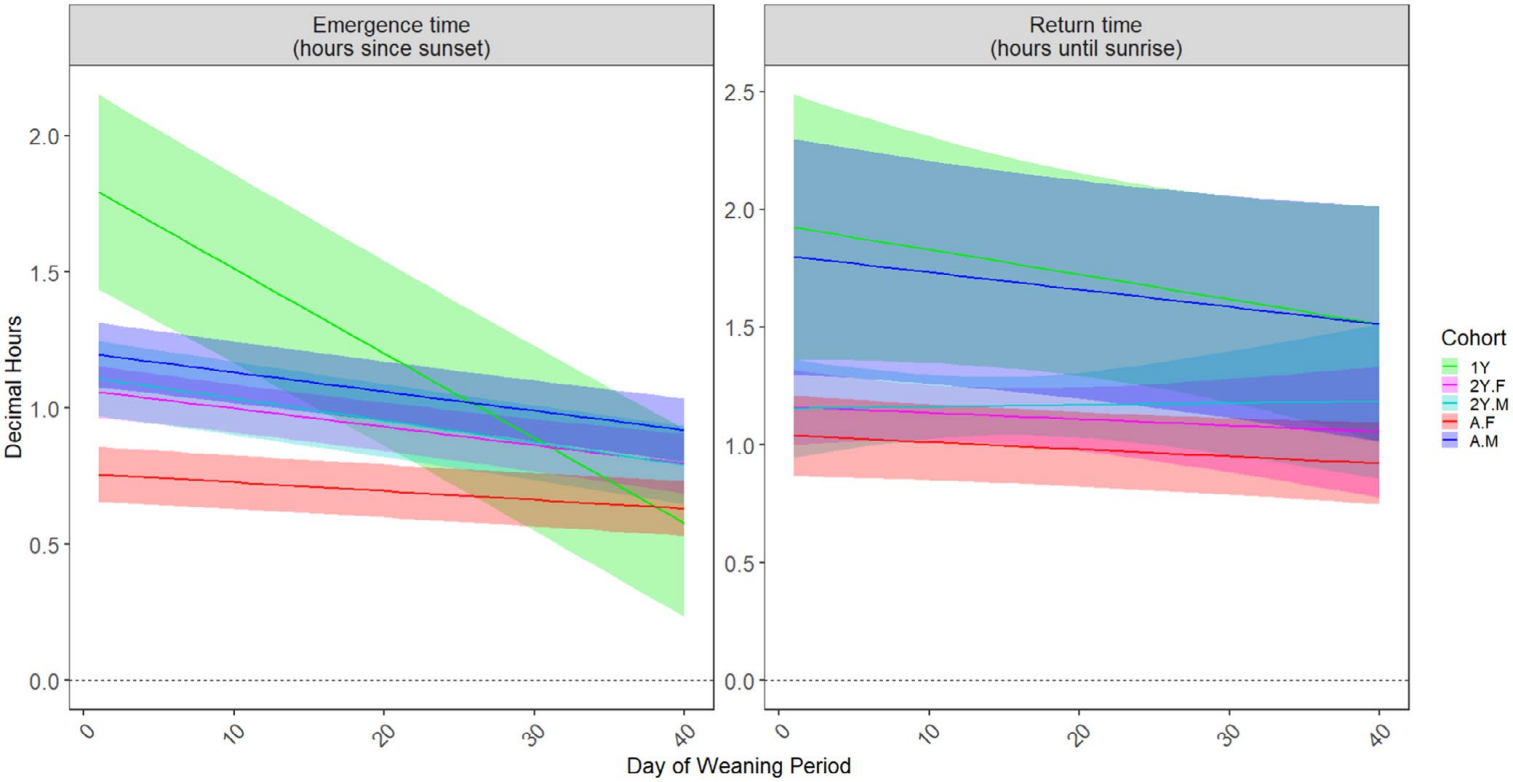
Modelled emergence (left) and return times (right) of Southern Bent‐wing Bats at the maternity roost during the weaning period. Day of weaning represents the numerical day of the weaning period defined in Table 1, and age or age‐sex cohort is denoted on the right (e.g., A.F = adult female). Juveniles (both sexes combined) are shown in green.

After recently commencing flying, in the weaning period, first‐year cohorts emerged latest (1YF: 63.6 m [55 m, 72.1 m], 1YM: 63.8 m [55.5 m, 72.3 m]) and returned earliest (1YF: 223.2 m [187.8 m, 258 m], 1YM: 219 m [184.2 m, 253.8 m]) (Figure 2 and Table S3). Throughout the 40‐day weaning period, first‐year bats initially emerged late, but they steadily emerged earlier by an estimated 1.9 m with each day of the weaning period (Table S2 and Figure 3). By the end of the weaning period, this age cohort was emerging at similar times to older cohorts (Figure 3). All age‐sex cohorts emergence times followed linear relationships with day of weaning (*p* < 0.050); however, older cohorts' emergence times showed much less change than first‐year cohorts throughout this 40‐day period (Table S2 and Figure 3). These patterns were not apparent for return times (Figure 3).

#### 3.2.2 Non‐Maternity Roost

**FIGURE 4:**
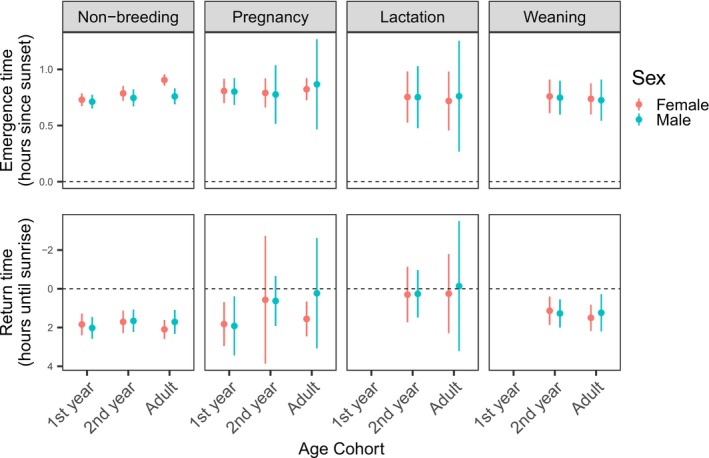
Estimated mean emergence and return times of Southern Bent‐wing Bat cohorts in each key reproductive period at the non‐maternity roost, predicted by peak emergence and return time models. Line length indicates 95% confidence intervals for each estimate. First‐years are reclassified as second‐years at the beginning of the following lactation period, so there are no first‐year individuals during this period at either roost. Weaning occurs at the maternity roost, and so first‐year individuals are not recorded during the weaning period at the non‐maternity roost.

At the non‐maternity roost, each cohort's emergence and return times did not significantly differ between periods, and rarely differed between cohorts (Figure 4 and Table S5). Statistically significant differences between cohort emergence times were only found during the non‐breeding period (Table S6), when occupancy peaks at this site (van Harten et al. 2022b). During the non‐breeding period, adult females (60.4 m, [56.1 m, 66.5 m]) emerged later than all other age‐sex cohorts (Figure 4). In this period alone, adult females emerged after adult males (51.7 m, [46.9 m, 56.5 m]).

### 3.3 Overnight Activity Patterns

**FIGURE 5:**
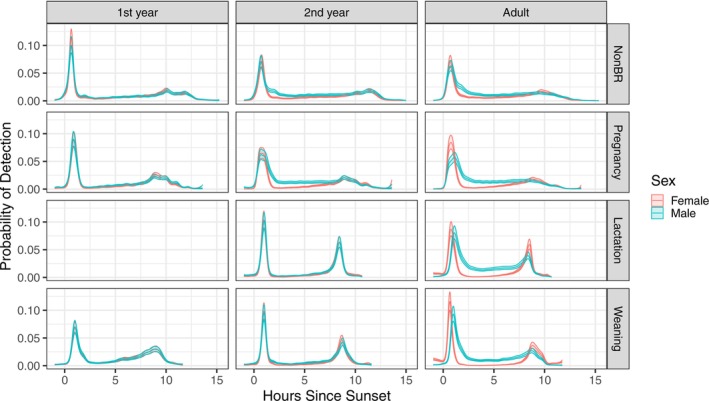
Modelled activity patterns throughout an average night in each key reproductive period at the maternity roost from an hour before sunset to an hour after sunrise. The band width represents 95% confidence intervals for each estimate. First‐years are reclassified as second‐years at the beginning of the lactation period, so first‐year cohorts are not included for that period.

Modelling the probability of detection by the PIT‐tag reader at the maternity roost throughout the night revealed variation between age and sex cohorts (Figure 5). In the pregnancy, lactation, and weaning periods, adult males were more likely than adult females to be within the cave between emergence and return peaks (i.e., adults males returned to the cave throughout the night more frequently than adult females) (Figure 5).

During the lactation period at the maternity roost, adult females were unlikely to return to the roost in the first few hours after initial emergence. However, their likelihood of detection increased incrementally 5 h after sunset until 7–8 h after sunset, when a return peak occurred (Figure 5). During the weaning period, adult females were unlikely to return to the roost until 6–7 h after sunset (Figure 5).

First‐year cohorts had relatively short foraging periods during the weaning period, characterised by later emergence, earlier return, and relatively high activity at the maternity roost overnight (Figure 5). The second‐year cohort's estimated nightly activity patterns were similar during lactation and weaning, comparable to those of adult females (Figure 5). Noticeable differences between male and female activity patterns commenced in the pregnancy period for second‐year bats (Figure 5), after which the behaviour of second‐year males and females began to resemble that of adult females. In the pregnancy period, second‐year females were less likely to return to the roost overnight than males; males were more likely to emerge later and females more likely to return later (Figure 5).

At the non‐maternity roost, there were very few differences in the probability of detection between the sexes of each age cohort, particularly in the non‐breeding period (Figure S1). Return peaks were strongest during the lactation and weaning periods, but otherwise, cohorts appeared to return in a more gradual manner throughout the night (Figure S1) when compared with the maternity roost (Figure 5).

## 4 Discussion

Early emergence and late return by insectivorous bats can indicate high resource requirements necessitating longer foraging periods (Arndt et al. 2018; Duvergé et al. 2000). In this population of Southern Bent‐wing Bats, the emergence and return behaviour of adult females varied with the annual reproductive cycle, whilst changes in the behaviour of younger cohorts appear to be most influenced by developmental stage. Adult females behaved similarly to adult males during the non‐breeding period but modified their behaviour to emerge earlier and return later than other cohorts during the pregnancy, lactation, and weaning periods. In contrast, adult males' emergence and return times did not vary substantially between reproductive periods. First‐year cohorts emerged late and returned early during the weaning period but reversed this behaviour in the non‐breeding period, extending their foraging periods following weaning.

Adult females' emergence and return behaviour from pregnancy to weaning was consistent with the high resource requirements imposed by reproduction (Barclay 1994; Kurta et al. 1989). Adult females foraged for longest during the weaning period, coinciding with a seasonal reduction in survival of this cohort (van Harten et al. 2022a). Though the reproductive status of females in our study was not known, it is likely that the majority were reproductively active once mature (N. Bail in prep). The energetic requirements of lactation in bats are immense. For example, the Little Brown Bat, *Myotis lucifugus* requires 50% greater energy investment during lactation than during pregnancy (Kurta et al. 1987). For Southern Bent‐wing Bats, current resource availability may not suffice to meet the energetic requirements of a proportion of adult females, which could contribute to the reduced summer survival of this cohort (van Harten et al. 2022a). The later emergence times of adult females in the non‐breeding period result in shorter foraging periods and reduced winter activity, which may support the ‘thrifty female’ hypothesis, whereby females conserve energy and maintain body condition over winter to support spring pregnancy (Jonasson and Willis 2011). Notably, however, this subspecies remains reasonably active over winter (van Harten et al. 2022b), unlike many North American and European species whose behaviour is explained by this hypothesis.

Interestingly, lactating females in this population did not return to the maternity roost often during the night, and presumably primarily nursed their young diurnally. In many bat species, females return to maternity roosts overnight to nurse young (Lumsden et al. 2021; McCracken and Gustin 1991; Rydell 1993), particularly if their roost is close to their foraging grounds (Daniel et al. 2008; Nurul‐Ain et al. 2017). This behaviour is thought to benefit reproducing females by reducing milk load (Rydell 1993) and allowing surplus water to be channelled into milk instead of being expelled, thus conserving water and reducing the risk of dehydration after nursing (Henry et al. 2002). Possibly, the high humidity in the maternity roost reduces the threat of dehydration for Southern Bent‐wing Bats, who regularly drink from water droplets within the roost (Codd et al. 1999). Alternatively, females might avoid returning to the roost for the primary purpose of nursing overnight, which could incur additional energetic costs or limit foraging distances (Nurul‐Ain et al. 2017). Finally, the insulating properties of the warm, humid maternity roost (Baudinette et al. 1994; Dwyer and Hamilton‐Smith 1965) reduce the thermoregulatory expenditure of pups (Chruszcz and Barclay 2002) and the need for mothers to return to their young overnight to warm them, which may be a motivating factor for nocturnal visitation in cavity‐roosting bat species (Otto et al. 2016).

In contrast to adult females, adult males and juveniles were more active overnight within the maternity roost during the lactation period, which might reflect their role in maintaining suitable climatic conditions for unweaned pups. Southern Bent‐wing Bat pups are usually clustered in an aven or crevice in the maternity roost, where the heat and humidity generated by active bats is trapped and sustained (Baudinette et al. 1994; Dwyer and Hamilton‐Smith 1965), effectively reducing juveniles' basal energetic requirements, permitting growth and improving survival (Webber and Willis 2018). Adult males may use torpor more often than females during reproduction, as found for other bat species (Dietz and Kalko 2006; Lausen and Barclay 2003; Turbill and Geiser 2006; Wilde et al. 1999), and therefore have less energetic imperative for extended foraging trips.

Sexual maturation requires significant energetic investment for both male and female bats (Brunet‐Rossinni and Austad 2004; Cheng and Lee 2002; Entwistle et al. 1998; Papadatou et al. 2008). Maturation is thought to occur in the second year of a Southern Bent‐wing Bat's life, as in closely related subspecies (Dwyer 1966), and our findings support this assumption. We show that both second‐year cohorts forage for relatively long periods during lactation and weaning (summer), likely to satisfy the energy and nutrient requirements imposed by sexual maturation prior to copulation. In other polygamous bats, frequent use of torpor or insufficient resource acquisition can delay sexual maturation and reduce reproductive success (Komar et al. 2020). In Southern Bent‐wing Bats, second‐year cohorts appear to satisfy the resource requirements of sexual maturation by foraging for extended periods prior to mating. In the pregnancy period, adult females' emergence times differ from all cohorts except second‐year females. This could indicate that a proportion of second‐year females could be facing similar energetic constraints to gestating adult females.

During the weaning period, the emergence and return behaviour of first‐year bats in our study is indicative of low foraging requirements, whilst resource needs are being partially met by maternal milk. As juveniles learned to fly, they emerged from the roost very late (almost 2 h after sunset) and returned early, suggesting that their foraging trips were relatively short due to a lower requirement for dietary nutrition from insect prey (Duvergé et al. 2000). Since predators can have great success preying on newly volant bats, particularly near dusk (Lee and Kuo 2001; Petrželková et al. 2004), late emergence in this age‐cohort could reduce predation risk. Typically, as bats wean, foraging activity increases while milk intake gradually reduces (Lučan 2009; Stern et al. 1997). Our results demonstrate progressively earlier emergence by first‐year bats as they are weaned, which is consistent with an increasing requirement for foraging in this cohort. Furthermore, this could allow these young bats to improve their flight skills in a ‘safer’ manner by emerging later when their flight competency is poorest, thus increasing their chance of later survival (Audet 1990).

The Southern Bent‐wing Bat has a temperate distribution and relies on seasonal peaks in food availability to reproduce and survive (Richardson 1977). Our results suggest that current resource availability is foremost limiting for reproducing females, who spend the most time outside the roost and emerge earlier than all other cohorts during pregnancy, lactation, and weaning and are therefore likely exposed to higher light levels and associated predation risk (Speakman 1995; Arndt et al. 2018). Theoretically, longer foraging periods will increase the risk of exposure to a range of threats, including entanglement and collisions with infrastructure, vehicles, or wind turbines (Department of Environment, Land, Water and Planning 2020; Bennett et al. 2022). Future reductions in resource availability (e.g., from drought or climate change) might lead to earlier emergence and longer foraging trips (Frick et al. 2012) with more associated risk, which could compound the impact of lower food availability, reducing survival and fecundity for these bats (Adams 2010; van Harten et al. 2022a).

PIT monitoring systems allow for the identification of cohort‐specific patterns, which is not possible with classical population‐level monitoring methods. The high fidelity of Southern Bent‐wing Bats to their maternity and non‐breeding roosts provides a unique opportunity to monitor large sample sizes of bats for extended periods using this technology. In our study, the location of the PIT reader at a constriction within the maternity roost (about 100 m from the entrance) allows for efficient detection of these fast‐flying bats (van Harten et al. 2019) but introduces uncertainty into our estimation of emergence and return times. However, although calculated emergence and return times differ between the PIT reader and *N2* monitoring at the cave entrance, our data suggest patterns in the order of cohort emergence peaks appear consistent across the two systems. Differences between estimates from the PIT and *N2* systems may be influenced by light sampling behavior where bats pass through the PIT‐tag reader but then spend some time near the entrance before emergence in order to discern external light levels (Fure 2006). The use of a single PIT reader at each cave means that we cannot infer whether observed bats are entering or exiting the roost. More detailed information could be obtained by installing additional antennae at each roost to facilitate determining the direction of flight.

Obligate cave roosting bats face unique challenges to their conservation, with a limited number of suitable roosts that take a long time to form and are becoming increasingly disturbed or otherwise made inaccessible to bats with urbanisation (e.g., collapsed by infrastructure, filled with rubbish, high levels of disturbance) (Tanalgo et al. 2022). This means that cave‐dwelling bats like the Southern Bent‐wing Bat are less capable of expanding their range in response to a changing climate, loss of foraging habitat, or in the occurrence of a catastrophic event. The large‐scale landscape changes and historic guano mining (and associated cave modification) (Hamilton‐Smith 1998) at Southern Bent‐wing Bat roosts have likely reduced the number of suitable roosts for these bats (Baudinette et al. 1994), as well as degrading the quality of foraging habitat without the option of range expansion. The more limited roosts become, the more intraspecific competition for species‐specific foraging resources increases for these bats, impacting the foraging period and emergence time for the most seasonally vulnerable cohorts (Duvergé et al. 2000). Relatively large congregations of bats at roosts can also facilitate predator attraction and can result in opportunistic predators having a significant impact.

Monitoring a population's emergence and return times allows for prompt detections of behaviour changes which are indicative of a reduction in resource availability or increase in resource requirements. PIT monitoring systems allow for the most affected cohorts to be identified, as the seasonal resource requirements of all cohorts within a population are not equal, as seen in our study. Efforts to reduce foraging pressures on the most at‐risk demographics can be concentrated on these demographics by provision of food or water for bats emerging early—for example, by using a UV light to concentrate resources during early emergence (Frick et al. 2023). Improving resource availability for lactating females in periods of drought, will improve recruitment for the population (Bourne and Hamilton‐Smith 2007) and can improve the subspecies' trajectory.

This long‐term PIT monitoring program provides a case study for how detection data can be applied and collated to explore resource partitioning in bat populations, guiding conservation efforts and providing insights into behavioural and ecological patterns, without causing additional disturbance (as opposed to trapping or in‐roost monitoring), which can be a significant issue when monitoring cave‐dwelling bats (Tanalgo et al. 2022). This study provides an example of how passive monitoring of emergence and return behaviour in colonial bats provides a useful proxy of cohort‐specific resource requirements, which can be used to inform ecological and conservation‐focused research and management.

## Author Contributions

**Nicola J. Bail:** conceptualization (lead), data curation (equal), formal analysis (equal), methodology (lead), writing – original draft (lead), writing – review and editing (equal). **Lindy F. Lumsden:** conceptualization (supporting), methodology (supporting), project administration (equal), resources (equal), supervision (equal), writing – review and editing (equal). **Terry Reardon:** conceptualization (supporting), investigation (equal), methodology (equal), resources (equal), writing – review and editing (supporting). **Emmi van Harten:** data curation (equal), methodology (equal), project administration (equal), validation (equal), writing – review and editing (equal). **Paul Clissold:** data curation (equal), formal analysis (supporting), software (equal), validation (equal), writing – review and editing (supporting). **Thomas A. A. Prowse:** conceptualization (equal), data curation (equal), formal analysis (equal), investigation (equal), methodology (equal), supervision (lead), writing – review and editing (lead).

## Disclosure

Statement of Inclusion: Our study involves a collaboration between multiple research institutions in Australia and uses data and input from multiple research and volunteer‐run projects. This study only involved Australian authors, but it builds on international emergence and return time theory. The authors acknowledge that this work was created on unceded land.

## Conflicts of Interest

The authors declare no conflicts of interest.

## Supporting information


Data S1.

## Data Availability

The data that support the findings of this study are openly available on the Dryad Digital Repository https://doi.org/10.5061/dryad.31zcrjdzn.
