# Supplementary material for: Emergence and Return Times in a Colonial, Cave‐Dwelling Bat: Age and Sex Differences Driven by Reproductive Cycle
Source: Ecol Evol. 2025 May 4;15(5):e71281. doi: 10.1002/ece3.71281 (PMC12050262; doi:10.1002/ece3.71281)
Supplement: Supplementary file 1 — Data S1. [file ECE3-15-e71281-s001.docx]

**Emergence and return times in a colonial, cave-dwelling bat: age and sex differences driven by reproductive cycle**

**Supporting Information**

**Table S1:** Number of individuals in each cohort PIT-tagged during each year of tagging. Tagging events occurred in January-February each year, when juveniles born that season could be distinguished from adults/subadults.

|  | **Adult Female** | **Adult Male** | **Juvenile Female** | **Juvenile Male** |
| --- | --- | --- | --- | --- |
| **2016** | 243 | 176 | 271 | 282 |
| **2017** | 188 | 161 | 292 | 358 |
| **2018** | 255 | 222 | 269 | 249 |
| **2022** | 109 | 112 | 137 | 138 |
| **Total** | 795 | 671 | 969 | 1027 |

**Table S2:** Goodness of fit statistics (R^2^ and % deviance explained) for emergence and return time models, and the relationships between day of weaning period and emergence or return time for each cohort. * indicates the relationship between the response variable and day of weaning period are statistically significant (p < 0.0001).

| **Cohort** | **Response** | **R^2^** | **DE (%)** | **Slope (day of weaning)** |
| --- | --- | --- | --- | --- |
| First-year bats | Emergence | 0.462 | 47.2 | -0.0312 * |
| Second-year females | Emergence | 0.356 | 38.7 | -0.00677* |
| Second-year males | Emergence | 0.522 | 54.2 | -0.00815* |
| Adult females | Emergence | 0.263 | 28.7 | -0.00324* |
| Adult males | Emergence | 0.244 | 26.6 | -0.00709* |
| First-year bats | Return | 0.0376 | 5.27 | -0.0105 |
| Second-year females | Return | 0.00333 | 2.43 | -0.00269 |
| Second-year males | Return | -0.0107 | 0.0168 | 0.000785 |
| Adult females | Return | 0.119 | 14.5 | -0.00301 |
| Adult males | Return | 0.0855 | 13.5 | -0.00734 |

**Table S3:** Statistically significant differences in estimated mean emergence time between cohorts in each reproductive period for Southern Bent-wing Bats, at the Naracoorte maternity roost.

| **Period** | **Earlier Emergence** | **Later Emergence** | **P value** |
| --- | --- | --- | --- |
| Non-breeding | First Year Females | Adult Females | <0.0001 |
| Non-breeding | First Year Males | Adult Females | <0.0001 |
| Non-breeding | First Year Females | Adult Males | <0.0001 |
| Non-breeding | First Year Males | Adult Males | <0.0001 |
| Non-breeding | First Year Females | Second Year Males | 0.0001 |
| Non-breeding | First Year Males | Second Year Males | 0.0012 |
| Pregnancy | Adult Females | Adult Males | <0.0001 |
| Pregnancy | Adult Females | Second Year Males | <0.0001 |
| Pregnancy | Adult Females | First Year Females | <0.0001 |
| Pregnancy | Adult Females | First Year Males | <0.0001 |
| Pregnancy | Second Year Females | Adult Males | 0.0001 |
| Lactation | Adult Females | Second Year Females | <0.0001 |
| Lactation | Adult Females | Second Year Males | <0.0001 |
| Lactation | Adult Females | Adult Males | <0.0001 |
| Lactation | Second Year Females | Adult Males | <0.0001 |
| Lactation | Second Year Males | Adult Males | <0.0001 |
| Weaning | Adult Females | Adult Males | <0.0001 |
| Weaning | Adult Females | Second Year Males | <0.0001 |
| Weaning | Adult Females | Second Year Females | <0.0001 |
| Weaning | Adult Females | First Year Males | < 0.0001 |
| Weaning | Adult Females | First Year Females | < 0.0001 |
| Weaning | Adult Males | First Year Females | 0.0165 |
| Weaning | Adult Males | First Year Males | 0.0064 |
| Weaning | Second Year Females | First Year Females | 0.0014 |
| Weaning | Second Year Females | First Year Males | 0.0007 |
| Weaning | Second Year Males | First Year Females | 0.0002 |
| Weaning | Second Year Males | First Year Males | 0.0001 |

**Table S4:** Statistically significant differences in estimated mean return time between cohorts for each reproductive period, at the Naracoorte maternity roost.

| **Period** | **Earlier Return** | **Later Return** | **P value** |
| --- | --- | --- | --- |
| Non-breeding | Adult Females | Second Year Females | <0.0001 |
| Non-breeding | Adult Females | Second Year Males | <0.0001 |
| Non-breeding | Adult Females | First Year Females | <0.0001 |
| Non-breeding | Adult Females | First Year Males | <0.0001 |
| Non-breeding | Adult Males | Second Year Females | <0.0001 |
| Non-breeding | Adult Males | Second Year Males | <0.0001 |
| Non-breeding | Adult Males | First Year Females | <0.0001 |
| Non-breeding | Adult Males | First Year Males | <0.0001 |
| Pregnancy | Adult Males | Adult Females | <0.0001 |
| Pregnancy | Adult Males | Second Year Females | <0.0001 |
| Pregnancy | Adult Males | Second Year Males | <0.0001 |
| Pregnancy | Adult Males | First Year Females | <0.0001 |
| Pregnancy | Adult Males | First Year Males | <0.0001 |
| Pregnancy | Second Year Males | First Year Females | 0.0075 |
| Pregnancy | Second Year Males | First Year Males | 0.0023 |
| Lactation | Adult Males | Adult Females | <0.0001 |
| Lactation | Adult Males | Second Year Females | 0.0028 |
| Lactation | Adult Males | Second Year Males | 0.0011 |
| Weaning | Adult Males | Adult Females | <0.0001 |
| Weaning | First Year Females | Adult Females | <0.0001 |
| Weaning | First Year Males | Adult Females | <0.0001 |

**Table S5:** Significant differences in estimated mean emergence time at the non-maternity roost, between cohorts for Southern Bent-wing Bats. There were only significant differences between groups at the non-maternity roost during the non-breeding period.

| **Period** | **Earlier Emergence** | **Later Emergence** | **P value** |
| --- | --- | --- | --- |
| Non-breeding | Adult Males | Adult Females | <0.0001 |
| Non-breeding | Second Year Females | Adult Females | 0.0005 |
| Non-breeding | Second Year Males | Adult Females | <0.0001 |
| Non-breeding | First Year Females | Adult Females | <0.0001 |

**Table S6:** Statistically significant differences in estimated mean return time between cohorts for each period, at the non-maternity roost for Southern Bent-wing Bats. The only difference was during the pregnancy period.

| **Period** | **Earlier Return** | **Later Return** | **P value** |
| --- | --- | --- | --- |
| Pregnancy | Second Year Males | First Year Females | 0.0024 |


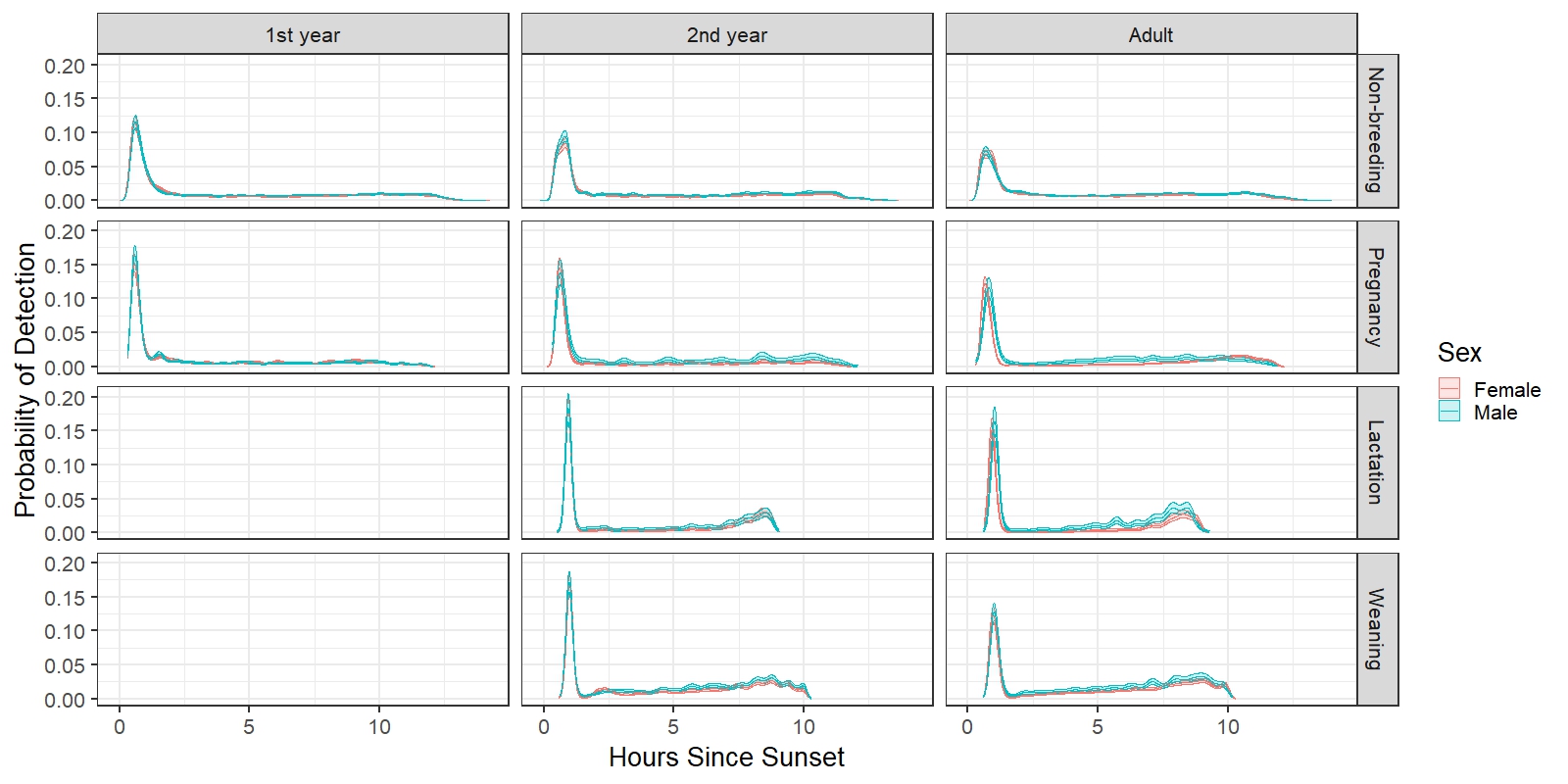
**Figure S1:** Modelled overnight activity patterns throughout an average night in each key reproductive period at the non-maternity roost for Southern Bent-wing Bats, where band width represents 95% confidence intervals for each estimate. First year cohorts are not included in the weaning and lactation periods as they are not active at the non-maternity roost during these periods.
